# Risk factors for knee replacement due to primary osteoarthritis, a population based, prospective cohort study of 315,495 individuals

**DOI:** 10.1186/1471-2474-15-217

**Published:** 2014-06-23

**Authors:** Hilde Apold, Haakon E Meyer, Lars Nordsletten, Ove Furnes, Valborg Baste, Gunnar B Flugsrud

**Affiliations:** 1 Orthopaedic department, Telemark Hospital, Skien, Norway; 2 Section for Preventive Medicine and Epidemiology, University of Oslo, Oslo, Norway; 3 Norwegian Institute of Public Health, Division of Epidemiolgy, Oslo, Norway; 4 Orthopaedic department, Oslo University Hospital, Oslo, Norway; 5 Faculty of medicine, University of Oslo, Oslo, Norway; 6 The Norwegian Arthroplasty Register, Department of Orthopaedic Surgery, Haukeland University Hospital, Bergen, Norway; 7 Department of Orthopaedic Surgery, Haukeland University Hospital, Bergen, Norway; 8 Department of Surgical Sciences, Faculty of Medicine and Dentistry, Bergen, Norway

**Keywords:** Osteoarthritis, Obesity, Knee joint replacement, Body mass index, Risk factor, Epidemiology

## Abstract

**Background:**

Osteoarthritis (OA) of the knee is a common and disabling condition. We wanted to investigate the modifiable risk factors Body Mass Index (BMI) and physical activity, using knee replacement (KR) as a marker for severely symptomatic disease, focusing on the interaction between these risk factors.

**Methods:**

315,495 participants (mean age 43.0 years) from national health screenings were followed prospectively with respect to KR identified by linkage to the Norwegian Arthroplasty Register. Data were analysed by Cox proportional hazard regression.

**Results:**

During 12 years of follow up 1,323 individuals received KR for primary OA. There was a dose–response relationship between BMI and heavy labour, and later KR. Comparing the highest versus the lowest quarter of BMI, the relative risk was 6.2 (95% CI: 4.2-9.0) in men and 11.1 (95% CI: 7.8-15.6) in women. Men reporting intensive physical activity at work had a relative risk of 2.4 (95% CI: 1.8-3.2) versus men reporting sedentary activity at work, the corresponding figure in women being 2.3 (95% CI: 1.7-3.2). The effect of BMI and physical activity at work was additive. The heaviest men with the most strenuous work had a RR of 11.7 (95% CI: 5.9-23.1) compared to the ones with the lowest BMI and most sedentary work. For women the corresponding RR was 15.8 (95% CI: 8.2-30.3). There was no association between physical activity during leisure and KR.

**Conclusion:**

We found that a high BMI and intensive physical activity at work both contribute strongly to the risk of having a KR. As the two risk factors seem to act independently, people with strenuous physical work with a high BMI are at particularly high risk for severely disabling OA of the knee, and should be targeted with effective preventive measures.

## Background

The etiology of osteoarthritis (OA) of the knee is multifactorial, and several studies and also meta-analyses have identified risk factors for the disease. Family history [1], age [2,3], ethnicity [4] and gender [5] are risk factors that make the joint more susceptible to the development of OA. Injury [6,7], physical activity [6,8-17], malalignment [18,19], smoking [6,20-26], and overweight [8,20,25,27-29] have also been well studied. BMI is one of the best documented risk factors for knee OA [8,20,25,27-29]. In a meta-analysis those who were overweight or obese had 2.96-times higher risk for knee OA than those with a normal weight (95% CI: 2.56-3.43) [25]. There is evidence that even weight within what is defined as normal according to the international classification of body-weight, do increase the risk of knee OA [30,31].

There has been reported conflicting evidence on the effect of smoking on OA. Two studies defining knee OA by x-ray changes or symptoms reported lower risk associated with smoking [6,20], and a recent study using joint replacement as a marker of OA found an inverse dose–response association between duration of smoking and joint replacement [32]. Other studies have found no association [22,23], and two meta-analyses have pointed out that the protective effect of smoking on incident OA was only apparent when pooling the results of case–control studies and cohort studies [24,25]. When restricting analyses to cohort studies the effect was no longer apparent. Hui et al. concluded that the negative association could be due to selection bias [24]. They noted that the use of controls recruited from hospitals is likely to include a higher proportion of smokers than the non-hospitalized population, and this could lead to a false negative association between smoking and OA. They also found that since smokers are normally thinner than non-smokers adjusting for BMI diluted the reported negative association between smoking and OA. In a more recent meta-analysis by the same group they investigated the effect of smoking on the progression of OA and made similar conclusions of no evidence of a protective effect of smoking on the progression of OA [26].

Occupational activity has been investigated by different occupations and by degree of work load. It has been reported a limited protective effect of sitting over 2 hours a day, and an increased risk in those kneeling and squatting excessively at work, and some occupations like farmers, construction workers, firefighters and floor-layers have been reported to have an increased risk of knee OA [6,8-11]. Meta-analyses have found evidence for a relationship between occupational activities involving kneeling, heavy lifting and knee OA, and that certain occupations like floor layers, and miners have an increased risk of knee OA [33,34].

Studies of physical activity at leisure and knee OA have shown some conflicting results [12-17]. Some studies have reported a protective effect on the joint [15], others no effect [13,17,35,36], and even others a detrimental effect on the knee joint [12,14,20]. A systematic review article from 2006 concluded that joint trauma was a greater risk factor than the sport itself and that the risk of OA was associated with the duration and intensity of the exposure [16]. Another systematic review from 2011, Urquhart et al. found that different measures of OA responded differently to physical activity. Physical activity was associated with an increase of osteophytes, but not with joint space narrowing, and also with some evidence of an increased cartilage volume measured with MRI. They suggested that even if physical activity lead to more osteophytes it may be beneficial to the health of the joint as a whole.

Some of the conflicting results when studying risk factor for OA may be due to the use of different criteria for OA identification, and differences in study designs. The use of radiographic defined OA for case identification has its limitation since the correlation between radiographic findings and patients symptoms is not that good [37]. Joint replacement is a proxy for severe OA, and even though it will only identify a few of all those affected by the disease it will identify those were OA has severe implications, both for the affected individual, and due to severe economic impact on the society. The non-prospective studies have limitations due to recall bias, and also evidence of a tendency to over-report height and to under-report weight [38].

Information on how risk factors interact would be of great value. Two independent case–control studies have reported the combination of a high BMI and physically demanding work, particularly work involving knee bending, to be particularly damaging to the knee joint [39,40].

With standardized measurements of height and weight, standardized questions on physical activity at both work, and at leisure we were able to investigate risk factors for severe OA in a large Norwegian cohort including both men and women, especially focusing on the interactions between these risk factors.

## Methods

### Population

Between 1985 and 1994 the National Health Screening Service (now part of the Norwegian Institute of Public Health) conducted population-based standardized cardiovascular health studies in all of Norway’s 19 counties [41,42]. In addition The University of Tromsø carried out a similar study in the city of Tromsø (Tromsø III study) between 1986 and 1987 [43]. The median participation rate in these studies was 75% (range: 55 to 88%). The purpose of all these studies was to investigate risk factors for cardiovascular disease.

By using the national 11-digit personal identification code we were able to link the data from the health screenings with the data on performed KR’s from the Norwegian Arthroplasty Register. The Norwegian Arthroplasty Register was established by the Norwegian Orthopaedic Association, and started to include information on KR’s from January 1994 [44]. The operating orthopaedic surgeon submits a standardised form to the register for each joint replacement performed. The form contains information about the diagnosis that led to the operation, the procedure, any previous KR, or other operation performed in the joint, and the type of implant used. Data on death and emigration was collected from Statistics Norway.

The exposure variables were collected from the health screenings performed between 1985 and 1994. The start of follow up was set to January 1^st^, 1994, the date the Norwegian Arthroplasty Register started registration of KR’s, and end of follow up was set to February 1^st^, 2006.

### Exclusion

We did not include individuals younger than 16 years at the screening (n = 484), or older than 80 years at start of follow up (n = 50). Individuals who had information in the register about revision surgery, but no information on primary surgery (n = 121), and individuals who according to Statistics Norway had died or emigrated before start of follow up (n = 4,076) were also excluded.

Of 320,226 individuals attending the health studies with complete information on the exposure variables, 315,495 (98.5%) were eligible for the study; 20,484 from the Tromsø II study, and 295,011 from the cardiovascular screenings in the 19 Norwegian counties.

### Exposure variables

The participants received a questionnaire with the invitation to the screening. They filled in the questionnaire at home and brought it to the screening where they had the opportunity to clarify misunderstandings with the study nurses. In all the screenings, body weight and height were measured at a consultation in a standardized way [45,46]. BMI was calculated as weight (in kilograms) divided by height (in meters) squared. Information on smoking habits was classified as; never smoker, former smoker, or current smoker. Information on physical activity at work and physical activity at leisure were each assessed in a four graded question classified as sedentary, moderate, intermediate or intensive (Table 1) [47]. The questions used to evaluate physical activity were first introduced in Sweden [47], and similar questions have been used by the World Health Organization in a study of trends and determinants in cardiovascular disease [48]. Both questions have been validated against maximum oxygen uptake during exercise [49]. The question concerning physical activity at leisure has been validated against maximum work capacity [43]. The question concerning physical activity at work has been validated against a 7-days diary [50].

**Table 1:** Questions used to classify physical activity at work and at leisure

**Physical activity at work**	
Sedentary	Predominantly sedentary, sitting (e.g. desk worker, watch maker, sitting assembly line worker (light goods))
Moderate	Sitting or standing, some walking (e.g. cashier, general office worker, light tool and machinery worker, foreman)
Intermediate	Walking, some handling of material (e.g. mailman, waiter, construction worker, heavy tool and machinery worker)
Intensive	Heavy manual labor (e.g. forestry worker, dock worker, farm worker, ditch digger)
**Physical activity at leisure**	
Sedentary	Reading, watching television or other sedentary activities
Moderate	Walking, bicycling or moving around in other ways at least 4 hours per week (including walking or cycling to place of work, Sunday walks etc.)
Intermediate	Participation in recreational athletics, heavy garden work etc. (note: duration of activity at least 4 hours a week)
Intensive	Participation in hard training or athletic competitions regularly and several times a week

### Statistical analyses

Cox proportional hazard regression method was used, calculating hazard ratios (hereafter called relative risks, (RR)) for having a KR. The event was defined as a participant’s first knee replacement (any side) for the diagnosis of primary OA during follow-up, either a total knee joint replacement with or without a patella button, or a medial unicondylar knee replacement. Censoring occurred for KR performed for other diagnosis than primary osteoarthritis, for death, for emigration, and at end of follow up. Survival time was calculated as the number of years from start of follow up to the time of event or censoring. We included the continuous variable age, the variables BMI and height categorized into quartiles, the categorical variables smoking habits, physical activity at work, and physical activity at leisure. The analyses were also performed with BMI and weight as continuous variables.

We divided the cohort into sex specific quartiles according to BMI and height and performed the analyses comparing the three highest quartiles to the lowest one (the reference quartile). The cohort was also divided into three groups according to World Health Organizations definition of normal weight (<25 kg/m^2^), overweight (25 kg/m^2^-30 kg/m^2^), and obesity (>30 kg/m^2^).

Population attributable risk (PAR) was calculated to estimate the possible reduction in KR’s if those exposed to higher levels of BMI and physical activity at work were able to shift their level of BMI and work activity to a lower level [51].

Log minus log curves confirmed that the proportional hazard assumption for the Cox model was fulfilled (data not shown). The analyses were performed by SPSS version 19 (SPSS Inc., Chicago, IL).

The study was approved by The Norwegian Data Protection Authority, and the Regional Committee for Medical and Health Research Ethics South East.

The numbers of included individuals in the tables may vary slightly due to some missing values.

## Results

153,795 men and 161,700 women were included in the study. The mean age for both sexes at screening was 43.0 years (SD 7.2), at start of follow up it was 46.8 years (SD 7.6), and at end follow up 58.8 years (SD 7.1). During 12 years of follow up, 1,323 individuals (0.42%) received their first KR due to primary OA. Of these 225 were unicondylar and 1,098 were total knee replacements (Table 2).

**Table 2:** Basic characteristics of the cohort of 315,495 Norwegian men and women

	**Men**	**Women**
No. of participants	153,795	161,700
No. of knee replacements for primary OA	430	893
BMI at screeening*	25.4 (3.2)	24.3 (4.0)
Age at screening years*	43.1 (7.2)	43.0 (7.3)
Age at start follow up years*	46.8 (7.6)	46.7 (7.6)
Age at operation*	62.8 (8.7)	64.4 (9.0)
Age at end follow up years*	58.7 (6.9)	58.9 (7.3)

*Mean (SD).

367 participants were censored because they received a KR for conditions other than primary OA. The most frequent indications for surgery in these cases were rheumatoid arthritis (n = 107) and sequela after meniscal injury (n = 81). A total of 19,690 individuals were censored because they died or emigrated during follow up.

In the multivariate Cox regression analyses the risk of KR increased with higher age at screening. The relative risk was 1.5 (95% CI: 1.4-1.5) for both men and women per five years increase. Women had double risk (RR 2.7, 95% CI: 2.3-3.3) compared to men for KR.

### Body mass index

**Figure 1:**
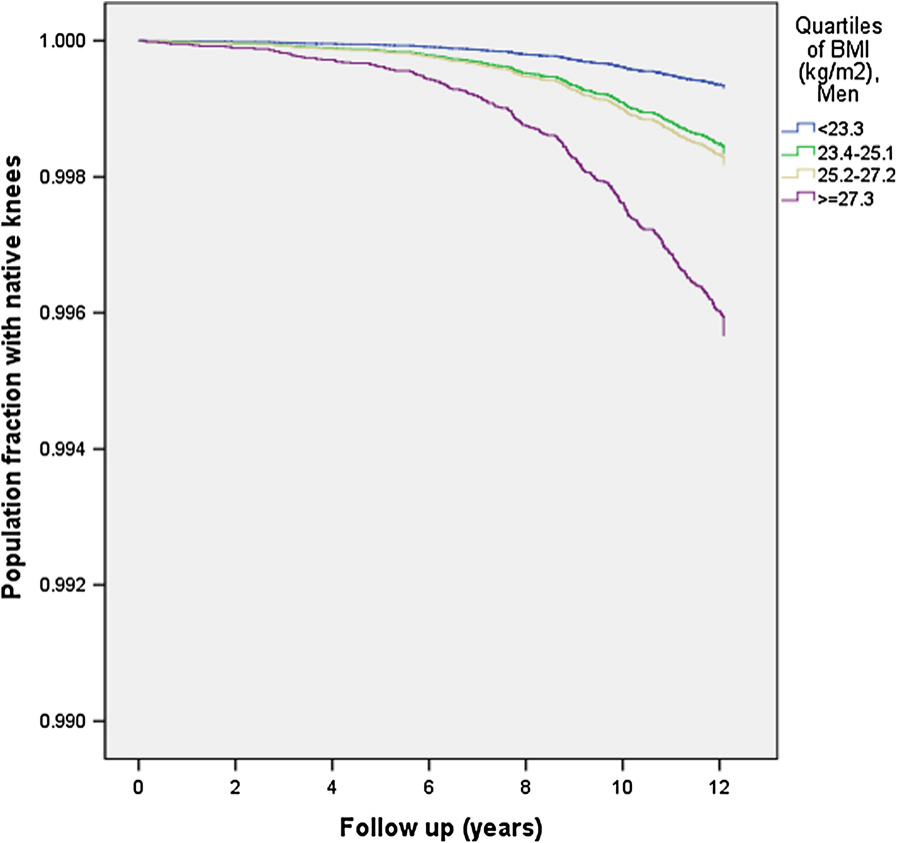
**Survival curve by quartiles of BMI for 153,795 male native knees undergoing their first KR due to primary OA in a Norwegian cohort.** Adjusted for age at screening, smoking habits, height, physical activity at work and at leisure time.

Men in the highest BMI quartile had more than six times the risk of KR compared to men in the lowest quartile of BMI (Table 3 and Figure 1).

**Table 3:** Crude rate and relative risk of KR due to primary OA in 153,795 Norwegian men

	**No. of participants**	**Person-years**	**No. of knee replacements**	**KRs per 10,000 person-years (crude rate)**	Multivariate adjusted RR* (95% CI)
***Body Mass Index, kg/m***^***2***^					
<23.3	38,967	470,734	32	0.7	1
23.4-25.1	38,099	460,094	79	1.7	2.35 (1.55-3.54)
25.2-27.2	38,918	469,847	93	2.0	2.58 (1.73-3.87)
> = 27.3	37,807	455,912	226	5.0	6.16 (4.23-8.95)
P (test for trend)					<0.0001
***Physical activity at work***					
Sedentary	58,430	705,549	114	1.6	1
Moderate	43,757	528,129	132	2.5	1.51 (1.18-1.95)
Intermediate	32,304	389,973	90	2.3	1.64 (1.24-2.17)
Intensive	19,118	230,737	94	4.1	2.41 (1.83-3.18)
P (test for trend)					<0.0001
***Physical activity at leisure***					
Sedentary	29,620	357,537	80	2.2	1
Moderate	80,762	974,895	245	2.5	1.02 (0.79-1.31)
Intermediate	38,982	470,681	98	2.1	0.96 (0.71-1.30)
Intensive	4,350	52,544	6	1.1	0.84 (0.36-1.94)
P (test for trend)					0.668
***Height***					
< 174	44,106	532,390	126	2.4	1
175-178	36,438	439,896	111	2.5	1.38 (1.06-1.78)
179-182	35,289	426,040	91	2.1	1.33 (1.01-1.76)
> = 183	37,960	458,286	102	2.2	1.64 (1.25-2.15)
P (test for trend)					0.001
***Smoking***					
Never smoker	46,945	566,770	126	2.2	1
Former smoker	41,371	499,259	161	3.2	1.00 (0.79-1.27)
Smoker	65,479	790,606	143	1.8	0.82 (0.64-1.04)
P (test for trend)					0.092

*Adjusted for age at screening, height, smoking habits, BMI and physical activity at work, and at leisure time.

**Figure 2:**
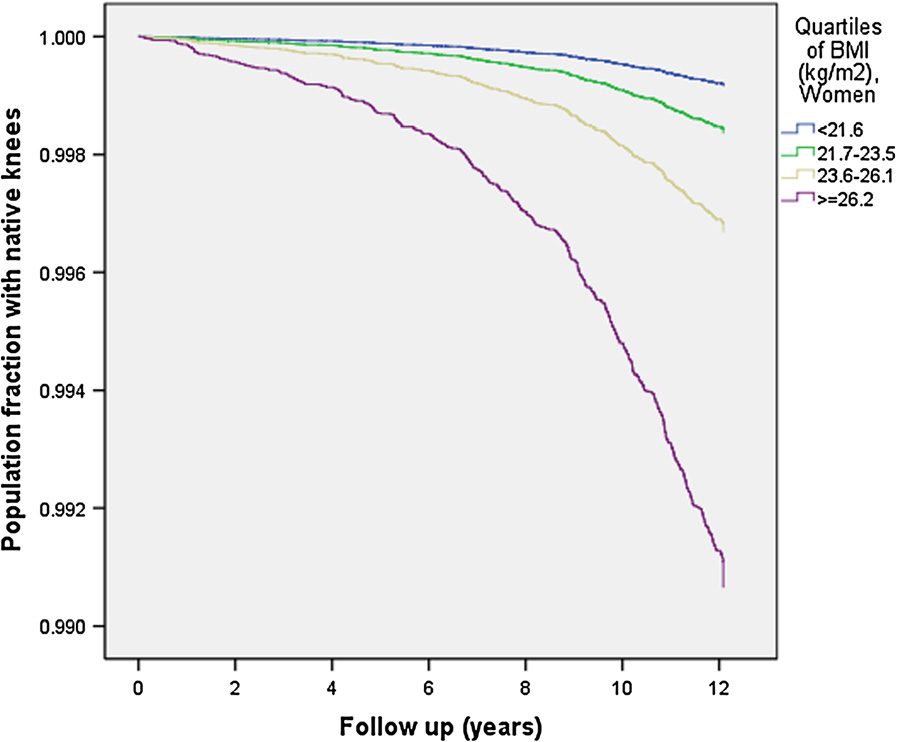
**Survival curve by quartiles of BMI for 161,700 female native knees undergoing their first KR due to primary OA in a Norwegian cohort.** Adjusted for age at screening, smoking habits, height, physical activity at work and at leisure time.

For women the risk of KR was more than eleven times higher in the highest compared to the lowest BMI quartile (Table 4 and Figure 2).

**Table 4:** Crude rate and relative risk of KR due to primary OA in 161,700 Norwegian women

	**No. of participants**	**Person-years**	**No. of knee replacements**	**KRs per 10,000 person-years (crude rate)**	Multivariate adjusted RR* (95% CI)
***Body Mass Index, kg/m***^***2***^					
<21.6	40,526	489,541	35	0.7	1
21.7-23.5	39,562	477,775	73	1.5	1.93 (1.29-2.89)
23.6-26.1	40,691	491,052	177	3.6	3.92 (2.72-5.64)
> = 26.2	40,911	491,705	608	12.4	11.06 (7.83-15.62)
P (test for trend)					<0.0001
***Physical activity at work***					
Sedentary	46,050	555,499	201	3.6	1
Moderate	84,368	1,017,358	498	4.9	1.18 (1.00-1.40)
Intermediate	27,744	334,751	149	4.5	1.30 (1.05-1.61)
Intensive	3,343	40,230	45	11.2	2.29 (1.65-3.18)
P (test for trend)					<0.0001
***Physical activity at leisure***					
Sedentary	31,322	377,346	190	5.0	1
Moderate	113,127	113,127	624	55.2	0.99 (0.84-1.16)
Intermediate	16,204	16,204	75	46.3	0.92 (0.70-1.21)
Intensive	968	11,687	4	3.4	1.72 (0.64-4.65)
P (test for trend)					0.797
***Height***					
< 161	44,123	531,632	325	6.1	1
162-165	42,320	510,399	238	4.7	1.10 (0.93-1.30)
166-169	38,461	464,179	150	3.2	0.93 (0.77-1.14)
> = 170	36,791	443,925	180	4.1	1.48 (1.23-1.80)
P (test for trend)					0.003
***Smoking***					
Never smoker	65,929	794,420	526	6.6	1
Former smoker	31,174	376,055	163	4.3	0.81 (0.68-0.96)
Smoker	64,597	779,720	204	2.6	0.66 (0.56-0.78)
P (test for trend)					<0.0001

*Adjusted for age at screening, height, smoking habits, BMI and physical activity at work, and at leisure time.

The association between body weight (kg) and KR was equally strong as that between BMI and KR (Additional file 1).

Dividing the cohort into groups according to the World health Organization’s (WHO) definition of normal weight, overweight and obesity gave similar results. Men with a BMI over 30 kg/m^2^ had a RR of 5.2 (95% CI: 4.0-6.9) compared to men with a BMI below 25 kg/m^2^. Women with a BMI over 30 kg/m^2^ had a RR of 8.7 (95% CI: 7.3-10.4) compared to women with a BMI below 25 kg/m^2^.

When entering BMI and weight as continuous variables, the sex difference in the risk estimates disappeared. Both men and women had a relative risk of 1.3 per 5 kilos of weight (men; RR 1.29 (95% CI: 1.25-1.34), women; RR 1.32 (95% CI: 1.29-1.34)), and a relative risk of 1.2 per unit of BMI (men; RR 1.18 (95% CI: 1.16-1.21), women; RR 1.16 (95% CI: 1.15-1.17)).

### Physical activity

Men had a dose–response relationship with increased risk for KR for all activity levels at work compared to sedentary activity (Table 3). Women with intermediate and intensive physical activity at work had an increased risk for KR compared to those with a sedentary activity level, whereas the risk in women with moderate physical activity was borderline significant (Table 4).

There were no associations between level of physical activity at leisure and the risk of later KR neither in men nor in women (Tables 3 and 4).

### Height

In both men and women those in the tallest quartile had around 50% increased risk for KR compared to those in the lowest quartile (Tables 3 and 4).

### Smoking

We did not find any significant association between smoking and the risk of KR in men (Table 3). Compared to female never smokers, the risk of KR were 34% lower in female smokers, and 19% lower in female former smokers (Table 4).

### The combined effect of physical activity at work and BMI

We also analysed the combined effect of having a high BMI and a high level of activity at work (Table 5). For men in the highest quartile of BMI with intensive physical activity at work there was an 11 fold increase in the risk compared to men in the lowest quartile of BMI with a sedentary physical activity at work. For women the corresponding risk increase was about 16. We tested for possible interaction between high BMI and physical activity at work using an interaction term. The interaction term for BMI and physical activity at work did not reach significance (p = 0.62).

**Table 5:** Relative risks* for KR due to primary OA at different levels of BMI and physical activity at work

**Physical activity at work**				
**Body mass index, kg/m**^**2**^	**Sedentary**	**Moderate**	**Intermediate**	**Intensive**
**Men**				
<23.3	1	1.2 (0.5-2.9)	1.2 (0.4-3.3)	1.9 (0.7-5.3)
23.4-25.1	2.0 (0.9-4.2)	2.6 (1.2-5.4)	3.5 (1.6-7.6)	5.1 (2.4-11.3)
25.2-27.2	1.2 (0.5-2.8)	4.1 (2.1-8.3)	3.7 (1.7-7.8)	5.6 (2.7-12.0)
> = 27.3	5.9 (3.0-11.4))	7.3 (3.7-14.2)	8.1 (4.1-16.2)	11.7 (5.9-23.1)
**Women**				
<21.6	1	0.6 (0.3-1.3)	0.8 (0.3-2.3)	3.1 (0.7-13.8)
21.7-23.5	1.4 (0.7-2.8)	1.7 (0.9-3.1)	1.3 (0.5-3.0)	2,6 (0.6-11.3)
23.6-26.1	2.8 (1.5-5.2)	3.2 (1.8-5.6)	3.1 (1.6-6.0)	7.9 (3.5-17.7)
> = 26.2	7.2 (4.1-12.8)	9.0 (5.2-15.7)	10.6 (5.9-18.8)	15.8 (8.2-30.3)

*Adjusted for Age at screening, height, smoking habits, and physical activity at leisure (95% CI).

Calculating PAR we found that if men with a BMI equal or above the third quartile and with moderate or higher levels of physical activity at work could reduce their risk to the levels of those unexposed (BMI lower than the third quartile and sedentary physical activity at work) we could expect to reduce the number of KR with up to 32%. For women the corresponding percentage was 41%.

## Discussion

We have found strong dose–response associations between BMI and physical activity at work, and severely symptomatic OA of the knee. There was no association between physical activity at leisure and severe OA of the knee. Participants with both high BMI and strenuous work were at particularly high risk. The associations were apparent for both genders but women were at higher risk than men, and the risk increased with age.

### Strength and weaknesses

Both BMI and occupational physical activity have been well documented as risk factors in previous literature. Our study confirms previous findings on these associations in a large unselected cohort of men and women, and brings new information on how overweight and physical activity at work interacts.

We used joint replacement with either unicondylar- or total knee prosthesis, as a marker of OA. Thus, we identified participants who developed severely symptomatic OA, who wanted a joint replacement, and who did not have contraindications to surgery. This may have introduced a bias, as severe obesity is a relative contraindication to surgery, leading us to underestimate the effect of obesity on the risk of needing a KR.

During the period of follow up data from the Norwegian Arthroplasty Register shows an increasing number of KR’s performed in Norway; from 25.1 per 100,000 inhabitants in 1995 to 70.5 per 100,000 inhabitants in 2005 [52]. If one compares the age specific incidence rates of primary KR’s between Norway and Sweden, two countries that are thought to have similar population and health care system, the rates differ. In the age group 55 to 64 the age specific incidence is 239 per 100,000 inhabitants in Norway, compared to 330 per 100,000 inhabitants in Sweden [53]. The increase in the number of performed KR’s in Norway, and the lower numbers of performed KR’s in Norway compared to Sweden is probably explained by the fact that Sweden introduced KR as a routine procedure earlier than Norway.

Not every hospital in Norway performed the procedure in the early part of our follow up, and the indication for whom to operate may have changed as the procedure have become more common. It may be that the surgeons were more restrictive to operate on overweight and obese patients in the early part of follow up. This could lead to an underestimation of the association between overweight and KR.

The mean age at start of follow up was 47 years, and at end follow up 59 years. As the mean age at KR in Norway is 70 years there is a proportion of our cohort who will receive their KR after end of follow up [52]. This could be a bias since we know that persons with a high BMI tend to be younger when they receive their KR compared to persons of normal weight [54,55]. This could have led us to overestimate the effect of BMI on the total population. However, our results are comparable with previous studies making it unlikely that this bias account for a large part of our risk estimates. The possible effect on the other risk factors is less obvious.

The main strength of this study is that it involves a large unselected Norwegian cohort making the results generalizable. The mean age at screening was 43.0 years, an age were the incidence of knee OA is low [56], making it likely that the majority of the participants were without symptoms of OA at screening.

The health care system in Norway is publicly funded and thereby almost free of charge for the patients, and the majority of patients that are permanently or temporarily out of work are provided for by the public sick leave pension. Therefore we do not think that the participants’ socioeconomic status represents a significant confounder neither with regards to inequality in the provision of surgery, nor due to differences in seeking surgery.

We do not have complete information on KR performed before the start of follow up, and some may erroneously have been classified as not operated. However, the mean age of the participants at start of follow up was 46.8, and this is an age were only a small proportion of the population will have undergone joint replacement. The completeness in the reporting of primary knee replacements to the Norwegian Arthroplasty Register is estimated to be 99%, and the number of censorings that should have been events is probably small [57].

### Body mass index

In our study group a higher BMI was associated with increased risk for joint replacement in the knee throughout the range of measured BMI, including the interval defined by WHO as normal. This concurs with the strong association between BMI and OA of the knee that has been identified in previous investigations [13,20,27,58-62]. Authors have used varying definitions of OA such as pain of certain duration, radiographic changes, or joint replacement surgery. Studies using joint replacement surgery as end point have reported risks comparable to the risks in our population [28,59-62]. As we had access to other important risk factors we could determine that BMI is a risk factor across gender, age, and level of physical activity, showing a dose–response relationship throughout the population’s range of BMI.

Higher weight could be caused by inactivity resulting from pain in an osteoarthritic joint rather than extra weight leading to OA. In our investigation the mean time between the screening and the joint surgery was 17.2 years, and reverse causation is therefore unlikely. Other studies have similarly shown that obesity precedes the development of OA in the knee [27].

### Occupational activity

We found a dose–response relationship between occupational activity and KR in both men and women. Occupational activities involving high physical workload, or special activities like kneeling, climbing and squatting have been shown to increase the risk of developing knee OA in several studies [11,39,63,64].

### The combined effect of physical activity at work and BMI

In our cohort persons with both a high BMI and intensive physical activity at work were at particularly increased risk for KR surgery. These results concur with the results from two previous case–control studies; In the study by Coggon et al. including persons at waiting list for KR they found that those with a BMI ≥ 30 kg/m^2^ and occupational activity with prolonged kneeling and squatting had an OR of 14.7 (95% CI: 7.3-30.2) compared to those with a BMI < 25 kg/m^2^ and no occupational kneeling or squatting [39]. The study of Vrezas et al. of radiographic OA in 298 men also concluded with a multiplicative interaction mode for the combined effect of high BMI and kneeling/squatting at work [40]. In a prospective cohort study of symptomatic knee OA Martin et al. also found evidence for a multiplicative interaction between BMI and heavy lifting and the association with clinical OA [8].

We did not find evidence for an interaction between BMI and physical activity at work, and the effect of BMI is therefor the same for all levels of physical activity at work. These results strongly indicate that individuals with heavy labour should be advised to maintain a body weight within the normal range.

To estimate the possible benefit of prevention regarding the combined effect of BMI and physical activity at work we calculated the PAR percentage. If men and women with a BMI equal or above the third quartile and with moderate or higher levels of physical activity at work could reduce their risk to the levels of those unexposed (BMI lower than the third quartile and sedentary physical activity at work) we found that there is a potential for a reduction in the numbers of KR’s with 32% for men and 42% for women.

The screening question about work activity was not designed to evaluate knee problems. We were not able to explore which specific activity that was harmful to the knee joint. The participants were asked about their activity at work throughout the last 12 months leading up to the screening, and we had no further information on the duration of the activity level.

Using KR as end-point, we assume that participants undergo joint replacement surgery when the disease becomes severely symptomatic. This may indicate that the joint has reached a certain stage of pathology, but the time of surgery may also be influenced by the participants need for maintaining physical activity. Having a physically demanding job may force a person to apply for knee replacement surgery at a more moderate stage of joint pathology. In Norway the economic incentive for keeping on working after symptom onset is less than in some other countries, due to a generous public pension for the sick and disabled as mentioned above. Detailed information on life-time exposure to physical activity might further elucidate the matter: if strenuous physical activity early in life shows a stronger association with later knee replacement it would indicate that the level of joint pathology is the key factor triggering surgery. If physical activity later in life is more closely associated with KR it might indicate that the person’s need for maintaining physical activity after the onset of OA strongly influences the decision to undergo surgery.

### Physical activity at leisure

We did not find any effect of spending a lot of leisure time doing physical activity on later risk of KR. Other studies have had conflicting results. Some have found a relationship, but varying according with the type of physical activity. There seems to be an association with repetitive high impact sports like soccer, team handball and ice hockey, and the risk is strongly associated with previous joint injury [6,65,66]. Also studies of other intensive activities but without high impact on the joint like long distance skiing have shown an increased risk of knee OA [67]. In studies of normal exercise like jogging, gymnastics and swimming, the association appears less important [13,36,58,68]. Our results are comparable to the findings in a resent Swedish cohort study that found no association between physical activity at leisure and KR [13]. A large prospective cohort study that also investigated severe OA requiring KR reported a dose response relationship between increasing levels of physical activity at leisure and KR. Although the effect was statistically significant, it was clinically small [14]. This study has many similarities with our current study. The reason for the discrepancies in the results may be related to joint injury. We did not have information on previous trauma, but we censored those who received an arthroplasty secondary to previous meniscal, or ligamentous injury. The effect of physical activity at leisure may appear more prominent if one investigates those receiving an arthroplasty due to secondary OA. The study by Wang et al. did not adjust for trauma, and since this is a well documented risk factor it may have affected the results.

The screening questions about physical activity at leisure used in our study were designed to evaluate the effect on cardiovascular disease, and they did not collect information regarding the level of impact on the joint. It might be that the classification of activity was not sensitive enough to evaluate the effect of particular kinds of physical activity on later OA. In our cohort only 2.6% of the men and 0.5% of the women reported intensive physical activity at leisure (hard training or athletic competitions regularly and several times a week). These small numbers in the most active category may also be a reason that we do not find an effect. However our findings suggest that physical activity at leisure as performed by the major part of the Norwegian population does not increase the risk for later severe knee OA.

### Smoking

Female smokers had a lower risk of KR compared to never-smokers. However, since we use surgery with KR as an endpoint, and it may be that smokers had comorbidities that made them unfit for surgery leading to a falsely low number of KR’s in the smoking group caution should be made when interpreting these results.

### Height

Men and women in the tallest quartiles had an increased risk for later KR due to primary OA. Previous studies have similarly found an increased risk for OA of the hip among tall men and women [69]. A prospective cohort study of women reported an increased risk for KR in tall women [59]. The association may be explained by mechanical factors such as a longer weight arm for muscles working across the knee joint, rendering the joint more susceptible to wear and tear. Another possibility as proposed by Liu et al. is that nutritional factors in early life both affect adult height as well as bone development and mineralization and leads to a predisposition for OA in tall individuals.

## Conclusions

Our findings confirm previous reports on a strong dose–response relationship between BMI and KR due to primary OA. A higher BMI entailed increased risk for severely symptomatic OA of the knee also within the range of BMI defined as normal. A higher level of physical activity at work increased the risk for KR later in life. The combination of heavy labor and a high BMI was particularly hazardous to the knee joint, leading to a 12-fold increase in risk for knee replacement among men, and a 16-fold increase among women.

Preventive measures should be directed at weight loss and work ergonomics, and be particularly aimed at doubly exposed persons.

## Competing interests

The authors declare that they have no competing interests.

## Authors’ contributions

HA participated in the conception and design of the study, performed the analysis and the interpretation of the data, and drafted the manuscript. LN obtained funding. LN, HEM, GBF, OF were involved in the conception and design of the study. GBF and HM performed analysis and interpretation of the data. VB contributed with statistical expertise. All the authors revised the manuscript for important intellectual content, and read and approved the final version of the manuscript.

## Pre-publication history

The pre-publication history for this paper can be accessed here:

http://www.biomedcentral.com/1471-2474/15/217/prepub

## Supplementary Material

Additional file 1 Relative risk of KR due to primary OA according to quartile of weight. Click here for file
