# Supplementary material for: Risk factors for knee replacement due to primary osteoarthritis, a population based, prospective cohort study of 315,495 individuals
Source: BMC Musculoskelet Disord. 2014 Jun 23;15:217. doi: 10.1186/1471-2474-15-217 (PMC4081510; doi:10.1186/1471-2474-15-217)
Supplement: Additional file 1 — Relative risk of KR due to primary OA according to quartile of weight. [file 1471-2474-15-217-S1.docx]

| **Additional file 1.** Relative risk of KR due to primary OA according to quartile of weight. | | | | | | |
| --- | --- | --- | --- | --- | --- | --- |
|  | No. of participants | Person-years | No. of  knee replacements | | Events per  10 000  person-years | Multivariate adjusted  relative risk  (95 % CI)* |
| Weight,  kilogram |  | | | | | |
| **Men** |  |  |  |  |  |  |
| <73.0 | 40,543 | 489,737 | 45 | 0,9 | | 1 |
| 73.0-79.5 | 36,823 | 444,675 | 69 | 1,6 | | 1.9 (1.3-2.7) |
| 79.6-87.0 | 38,803 | 468,427 | 102 | 2,2 | | 2.7 (1.9-3.9) |
| >87.0 | 37,624 | 453,772 | 214 | 4,7 | | 6.5 (4.6-9.2) |
| **Women** |  | | | | | |
| <58.5 | 41,665 | 503,269 | 44 | 0,9 | | 1 |
| 58.5-64.5 | 41,015 | 495,208 | 105 | 2,1 | | 2.5 (1.7-3.5) |
| 64.5-72.0 | 40,800 | 492,234 | 193 | 3,9 | | 4.4 (3.1-6.1) |
| >72.0 | 38,215 | 459,422 | 551 | 12,0 | | 11.9 (8.7-16.3) |
| *Adjusted for age at screening, height, smoking habits, and physical activity at work, and at leisure time | | | | | | |
